# Systemic Pharmacological Approach to Identification and Experimental Verification of the Effect of Anisi Stellati Fructus Extract on Chronic Myeloid Leukemia Cells

**DOI:** 10.1155/2019/6959764

**Published:** 2019-12-12

**Authors:** Youn Sook Kim, Su Yeon Suh, Yong Tae Ahn, Chul Won Lee, Sang Yull Lee, Soon Cheol Ahn, Won G. An

**Affiliations:** ^1^School of Medicine, Pusan National University, Yangsan 50512, Republic of Korea; ^2^Okpo Korean Medicine Clinic, Daegu 42970, Republic of Korea; ^3^Research Institute for Korean Medicine, Pusan National University, Yangsan 50612, Republic of Korea; ^4^Department of Pharmacology, School of Korean Medicine, Pusan National University, Yangsan 50612, Republic of Korea

## Abstract

Anisi stellati fructus (ASF) is the dried fruit of the *Illicium verum* Hook.f. tree. The aim of this research was to evaluate the antileukemic effect of ASF on chronic myeloid leukemia (CML) cells, which was hypothesized from the systemic pharmacological analysis of ASF, focusing on the combined effect of ASF extract (ASFE) and imatinib (IM). The compounds of ASF were identified using the Traditional Chinese Medicine Systems Pharmacology database and analysis platform. The target gene information was acquired from the UniProt database. The compound and target interaction network was generated from Cytoscape 3.7.1. Using this analysis, 10 compounds effective against CML cells were obtained. ASFE was prepared and analyzed by high-pressure liquid chromatography to provide experimental proof for the relationship between ASF and CML. The anti-p210^Bcr-Abl^ effects of ASFE and ASFE + IM combination were evaluated by western blotting. Either ASFE alone or in combined treatment with IM on K-562 CML cells resulted in a significant reduction of the Bcr-Abl levels. As expected from the systemic analysis results, ASF had antileukemic activity, showing that it is a potential therapy for CML.

## 1. Introduction

Anisi stellati fructus (ASF) is fruit that comes from the evergreen aromatic tree, *Illicium verum* Hook. f. According to *The Great Pharmacopoeia* written by Li Shi Zhen, published in 1596, the fruit of *Illicium verum* Hook. f. (Chinese star anise) has been used as a remedy for infectious diseases. ASF has long been used as a food as well as for medical purposes because of its effect on eliminating odor and relieving symptoms such as high fever, diarrhea, and vomiting [1]. Additionally, Tamiflu, a treatment for swine-origin influenza A, was developed based on the components of ASF [2].

Chronic myeloid leukemia (CML) is considered to be a myeloproliferative neoplasm characterized by the expansion of a clone of hematopoietic cells that carries the abnormal Philadelphia chromosome and fused *Bcr-Abl* gene [3]. Bcr-Abl is a constitutively active cytoplasmic tyrosine kinase [4]. Bcr-Abl fusion protein is present in 95% of CML cases and 20%–30% of acute lymphoblastic leukemia cases [5, 6]. For these reasons, p210^Bcr-Abl^ is a major target of tyrosine kinase inhibitors (TKIs) [7]. CML is diagnosed in the chronic phase and is transformed into the acute phase in an average of 2-3 years, resulting in mortality within a few months despite antileukemic combination therapy [8, 9].

Imatinib (brand name: Gleevec, Novartis; formerly called STI571) is a relatively specific inhibitor of the Bcr-Abl tyrosine kinase, which has efficacy in CML [10]. Although survival from CML was increased by imatinib (IM), drug resistance by mutated forms of Bcr-Abl led to the development of new drugs such as dasatinib, nilotinib, bosutinib, and ponatinib [11]. The K-562 CML cell line is highly resistant to various stimuli. However, the development of new drugs often causes resistance to these drugs [12].

To date, some studies have shown a correlation between infection and cancer, and approximately 2 million emerging cancer cases are due to infections from microorganisms [13–15]. Numerous studies have shown that antimicrobial peptides have cytotoxic effects on cancer cells and have the possibility of being used in anticancer therapy either alone or in combination with other conventional drugs. An increasing number of studies have shown that some of the cationic antimicrobial peptides, which are toxic to bacteria but not to normal mammalian cells, exhibit a broad spectrum of cytotoxic activities against cancer cells [16, 17]. Our previous study has shown that ASF contains 49 identified compounds, of which 35 compounds were analyzed to have target genes related to antimicrobial activities [18]. However, there has been minimal research on the anticancer effects of ASF. Only ASF has been reported to inhibit the metastasis and angiogenesis of malignant cancer cells [19]. In the present study, the 49 identified compounds of ASF were further investigated for their efficacy against cancer using systemic pharmacological analysis. The results showed that 20% of the total ASF compounds were associated with CML. Based on this result, we characterized the antileukemic potential of ASF against K-562 CML cells. The results showed that the ASF extract (ASFE) either alone or in combination with IM significantly reduced the amount of p210^Bcr-Abl^ protein, suggesting further studies to confirm ASF as an effective antileukemic adjuvant.

## 2. Materials and Methods

### 2.1. Chemicals and Reagents

IM was purchased from Sigma-Aldrich (St. Louis, MO, USA). Dulbecco's phosphate-buffered saline (D-PBS) was purchased from Gibco (Grand Island, NY, USA). High-pressure liquid chromatography- (HPLC-) grade methanol and water were obtained from JT Baker (Phillipsburg, NJ, USA). The Cell Counting Kit-8 (CCK-8) was obtained from Dojindo Molecular Technologies (Rockville, MD, USA). Anti-Bcr and secondary antibodies were purchased from Cell Signaling Technology (Danvers, MA, USA). Antibody specific to beta-actin was purchased from Sigma-Aldrich. The 4-anisaldehyde, 4-prophenylanisole, and other chemicals were purchased from Sigma-Aldrich.

### 2.2. Systemic Pharmacological Analyses of ASF

#### 2.2.1. Identification of ASF Compounds

To search for identified compounds in ASF and to investigate the relationship between the compounds and cancers, the Traditional Chinese Medicine Systems Pharmacology (TCMSP, http://lsp.nwu.edu.cn/) database and analysis platform were used. The 49 compounds were found in ASF, and all diseases related to each compound were recorded. After screening a total of 49 compounds, 33 compounds were found to have relationships with cancer categories. Among them, 10 CML-related compounds were identified. To screen bioactive compounds of these 10 CML-related compounds, five parameters related to absorption, distribution, metabolism, and excretion were used: molecular weight (MW), oral bioavailability (OB), Caco-2 permeability (Caco-2 cells), drug likeness (DL), and drug half-life (HL) [20–22].

#### 2.2.2. Target Collection

Molecular targets of the 10 CML-related compounds and the official gene information were acquired and listed using the UniProt database (http://www.uniprot.org).

#### 2.2.3. Network Construction

To visualize the 10 CML-related compounds and their possible targets, a compound (C)-target (T) network was constructed using Cytoscape 3.7.1, an open-source bioinformatics platform for visualizing molecular interaction networks (https://cytoscape.org/). In the network, the node denoted either compounds or target proteins, and the edges represented compound-target connections.

### 2.3. Preparation of ASF

Dried fruits of ASF (1.0 g) obtained from Kwangmyungdang Medical Herbs (Ulsan, Republic of Korea) were broken into pieces, ground, and mixed with 10 mL of methanol. The mixture was vortexed and sonicated for 30 min at room temperature. The resulting mixture was centrifuged at 3,000 rpm for 30 min at room temperature. The supernatant was carefully collected and filtered through a 0.2 *μ*m syringe filter (BIOFACT™, Yuseong-Gu, Republic of Korea). The filtrate was evaporated using a pressured gas blowing concentrator (MGS-2200; EYELA, Chula Vista, CA, USA). The yield of the ASF was 5%.

### 2.4. Chemical Profiling of ASF by HPLC

#### 2.4.1. Chromatography Conditions

Analysis was performed using an Agilent 1290 HPLC system (Agilent Technologies, Palo Alto, CA, USA) consisting of a quaternary pump, an autosampler, a column oven, and a diode-array detector. The data were processed using ChemStation software, rev. B. 03. 02 (Agilent). To separate the sample, an ACQUITY UPLC BEH C18 column (2.1 × 100 mm, 1.7 *μ*m; Waters, Milford, MA, USA) was used. The mobile phase was comprised of 0.1% formic acid in distilled water (A) and acetonitrile (B). The column temperature was maintained at 40°C. The analysis was conducted at a flow rate of 1.0 mL/min with PDA detection wavelengths of 254 nm and 275 nm. The injection volume was 5 *μ*L.

#### 2.4.2. Preparation of Standard Solutions

The standard stock solutions of 4-anisaldehyde and 4-prophenylanisole were prepared in methanol and stored at 4°C. A working standard solution was prepared by serial dilution of stock solutions with methanol. All calibration curves were obtained from assessments of peak areas of standard solutions in the following concentration ranges: 4-anisaldehyde, 0.10–2.00 *μ*g/mL; 4-prophenylanisole, 0.10–2.00 *μ*g/mL.

#### 2.4.3. Preparation of Sample Solution

The resulting solution of ASFE was filtered through a 0.2 *μ*m syringe filter and injected into the HPLC instrument.

### 2.5. Cell Culture

Chronic myeloid K-562 leukemia cells were obtained from the American Type Culture Collection (Manassas, VA, USA) and maintained in RPMI 1640 containing 10% (v/v) heat-inactivated fetal bovine serum (Sigma-Aldrich) and 1% (v/v) penicillin/streptomycin (Gibco/BRL, Grand Island, NY) at 37°C in a 5% (v/v) CO_2_ incubator.

### 2.6. Cell Viability Assay

Cell viability was determined using the CCK-8 assay according to the manufacturer's instructions. K-562 cells were seeded at a density of 1 × 10^4^/well in a 96-well plate. After incubation for 24 h, the cells were treated with dimethyl sulfoxide or various concentrations of ASFE for 24 h at 37°C in an incubator with a 5% CO_2_ atmosphere. The cells were then incubated with 10 *μ*L of CCK-8 reagent for 4 h at 37°C. Absorbance was measured at 450 nm using an ELISA microplate reader (Tecan, Mannedorf, Switzerland). Data are presented as the percentage viabilities of untreated cells (100%). The assay was independently repeated three times.

### 2.7. Western Blot Analysis

After treatment with ASFE, IM, and ASFE + IM by either a dose-dependent or time-dependent manner, K-562 cells were harvested by centrifugation at 4°C, washed once with ice-cold D-PBS, resuspended in lysis buffer (50 mM Tris (pH7.5), 2 mM EDTA, 100 mM NaCl, and 1% NP-40) containing protease inhibitor cocktail (Sigma-Aldrich), and incubated for 30 min at 4°C. Following centrifugation for 15 min at 13,000 rpm at 4°C, the protein concentration of the lysate was determined using a bicinchoninic acid (BCA) Protein Assay Kit (Thermo Fisher Scientific, Waltham, MA, USA), according to the manufacturer's instructions. 30 *μ*g of total protein was separated on a 6% or 7% sodium dodecyl sulfate-polyacrylamide gel and electroblotted onto polyvinylidene difluoride (PVDF) membranes (Bio-Rad, Hercules, CA, USA). The blots were incubated with a blocking solution (5% skim milk) for 1 h at room temperature, followed by incubation overnight with the primary antibodies (anti-Bcr, 1 : 3,000 dilution; anti-beta-actin, 1 : 1,000 dilution) at 4°C. The blots were then washed three times with Tween-20/Tris-buffered saline (TTBS), followed by incubation with the appropriate horseradish peroxide-conjugated secondary antibodies (1 : 3,000 dilution) for 1 h at room temperature, and then washed with TTBS. Immunoreactive bands were visualized using the Enhanced Chemiluminescence Detection Solution (ECL Plus; Thermo Fisher Scientific). ImageJ software was used to quantitate the band densities.

### 2.8. Quantification of Apoptosis by Annexin V Labeling

Apoptosis was quantified using an annexin V and 7-AAD Kit (Millipore, Hayward, CA, USA) according to the manufacturer's instructions. Briefly, after 1 × 10^6^ K-562 cells were seeded in a 60 mm cell culture dish, ASFE (2 *μ*g/mL), IM (2.0 *μ*mol), or their combination was added. Following incubation for 24 h at 37°C in a 5% CO_2_ incubator, the cells were collected and incubated with annexin-V and 7-AAD for 20 min at room temperature in the dark. The events for live, dead, and early (annexin V+/7-AAD+) cells were counted using the Muse cell analyzer (Merck Millipore, Billerica, MA, USA).

### 2.9. Statistical Analysis

Statistical analysis was performed using SPSS statistical software for Windows, version 23 (IBM, Armonk, NY, USA). Data are expressed as the mean ± standard deviation (SD) of triplicate determinations. Differences in means between groups were subjected to one-way analysis of variance followed by the least-significant multiple comparison test and independent *t*-test. *P* values <0.05 were considered significant differences.

## 3. Results

### 3.1. Screening 33 Cancer-Related Compounds

According to the TCMSP database, ASF contained 49 identified compounds. Every compound and its related diseases were investigated, and 33 cancer-related compounds were screened (Table 1).

### 3.2. Collecting 10 CML-Related Active Compounds and Systemic Pharmacological Analyses

Ten CML-related compounds and the analyzed results are shown in Table 2. To search bioactive compounds, the criteria of each parameter were suggested. Molecular weights from 180 to 500 Daltons were recognized as comparatively more druggable [23]. Oral bioavailability (OB) ≥30% and drug likeness ≥0.18 were used as ideal drug characteristics [24, 25]. Caco-2 cell permeability was tested using an *in vitro* experiment with the Caco-2 human intestinal cell line. Drug half-life (*t*1/2) represented how long it took for the drug to be internalized and reduced by half; a drug half-life ≤4 h was classified as the fast elimination group, between 4 and 8 h as the midelimination group, and ≥8 h as the slow-elimination group [20, 21].

Among the 10 CML-related compounds, luteolin (MW = 286.25, OB (%) = 36.16, Caco-2 = 0.19, DL = 0.25, and HL = 15.94), kaempferol (MW = 286.25, OB (%) = 41.88, Caco-2 = 0.26, DL = 0.24, and HL = 14.74), and quercetin (MW = 302.25, OB (%) = 46.43, Caco-2 = 0.05, DL = 0.28, and HL = 14.4) largely met the suggested criteria and have been reported as representative antibacterial compounds in previous studies [26–28], including our study [18]. Additionally, luteolin exhibited an antibacterial action that suppressed the activity of bacterial DNA topoisomerases 1 and 2 and reduced the synthesis of nucleic acids and proteins [29] and was also known to have antioxidant, anticancer, anti-inflammatory, and neuroprotective effects [30]. Kaempferol is a flavonoid and possesses anti-inflammatory, antioxidant, anticancer, and antibacterial activities [31–33]. It has been shown to be effective against acne-induced *Propionibacterium acnes* and *Helicobacter pylori* found in the stomach [34]. Quercetin is a compound that plays an important role in inflammation, cancer, aging, cell signaling, proapoptotic effects, antiproliferative effects, antioxidant effects, and growth suppression [35, 36]. Astragalin (MW = 448.41, OB (%) = 14.03, Caco-2 = -1.34, DL = 0.74, and HL = N/A) has been reported to inhibit autophagosome formation in airways [37]. Honokiol (MW = 266.36, OB (%) = 60.67, Caco-2 = 1.43, DL = 0.15, and HL = 2.88) has been shown to have antitumorigenic, anti-inflammatory, and antioxidant effects [38–40]. (+)-Catechin (MW = 290.29, OB (%) = 54.83, Caco-2 = −0.03, DL = 0.24, and HL = 0.61) is a well-known phenolic compound found in tea and wine.

### 3.3. Network Construction of 10 CML-Related Compounds and Their Related Targets

**Figure 1:**
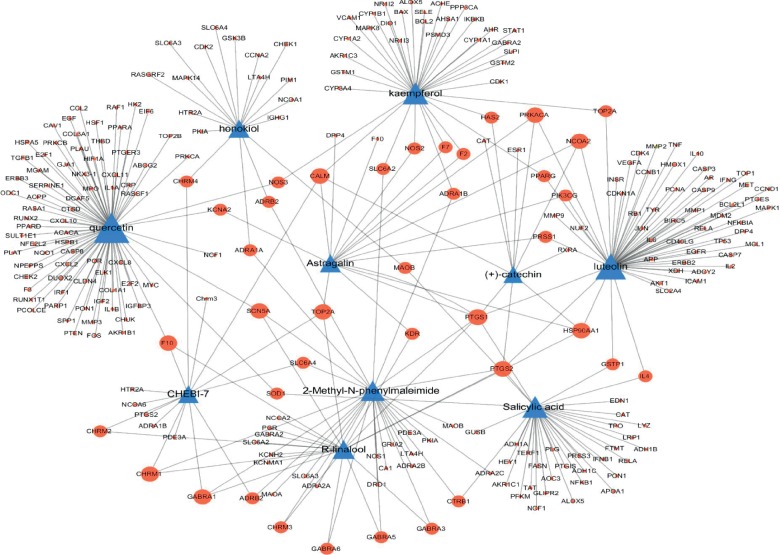
The network of 10 CML-related compounds and their related target genes. Triangle nodes represent compounds and circular nodes represent targets. Node size is relative to the degree, and the edges demonstrate the interaction between nodes.

To visualize the target genes of 10 CML-related compounds, a compound (C)-target (T) network was established. A total of 313 target proteins were collected from the TCMSP, and the official gene names of the targets were obtained from the UniProt database.  Figure 1 shows that the C-T network revealed interactions between 10 CML-related compounds and 313 target genes. The nodes showed the 10 CML-related compounds and the target genes, and the edges represented the interaction of compounds and targets. The size of nodes was related to the degrees. Quercetin (degree = 77) had the greatest interaction with targets, followed by luteolin (degree = 56), kaempferol (degree = 36), salicylic acid (degree = 35), 2-methyl-N-phenylmaleimide (degree = 29), *R*-linalool (degree = 20), honokiol (degree = 18), astragalin (degree = 16), CHEBI:7 (degree = 15), and (+)-catechin (degree = 10).

### 3.4. HPLC Analysis of ASFE

**Figure 2:**
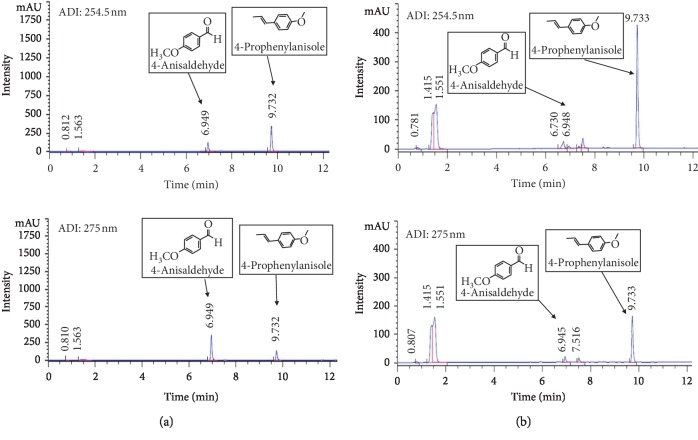
Chromatograms of two major components (4-anisaldehyde and 4-prophenylanisole) identified in Anisi stellati fructus extract (ASFE). (a) Chromatograms of the standard mixture. (b) Chromatograms of the major components in ASFE. The chromatograms were obtained at 254.5 and 275 nm.

The main components of ASFE were determined using an HPLC system to evaluate the quality of ASFE. Because 4-anisaldehyde and 4-prophenylanisole [(E)-anethole] have been reported to be the main compounds of ASFE, we used them as standards and conducted the analysis. As shown in the representative chromatographic fingerprint of ASFE, the main components of ASFE were 4-anisaldehyde and 4-prophenylanisole (Figure 2).

### 3.5. Effect of ASFE on the Cell Viability of K-562 Cells

**Figure 3:**
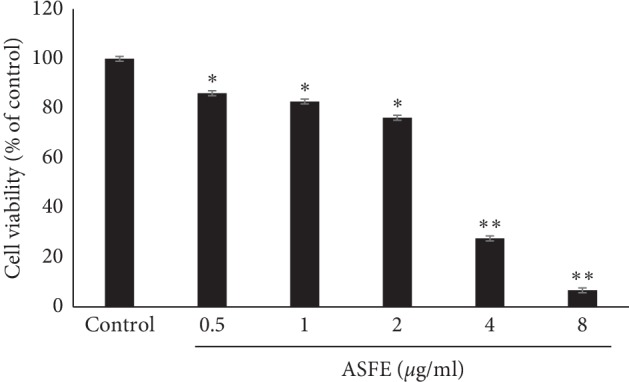
Cytotoxicity of the ASFE on K-562 cells. The cells (1 × 10^4^/well) were determined using a CCK-8 assay.

The possible cytotoxic effect of ASFE (0–8 *μ*g/mL) on K-562 cells was assessed (Figure 3). ASFE significantly inhibited the proliferation of K-562 cells in a dose-dependent manner. The IC50 value of ASFE was approximately 4.03 ± 2.3 *μ*g/mL (^*∗*^*P* < 0.05; ^*∗∗*^*P* < 0.01).

### 3.6. ASFE Treatment on K-562 Cells Decreases the Amount of p210^*Bcr-Abl*^ in a Time-dependent Manner

**Figure 4:**
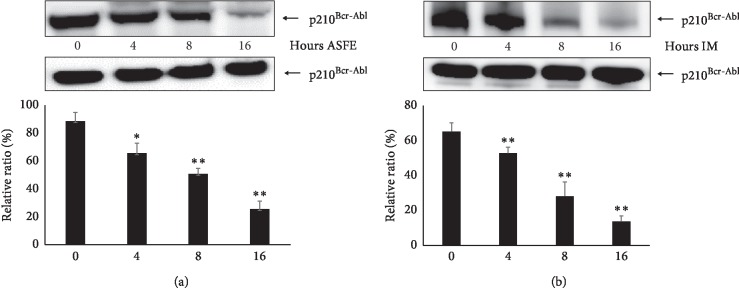
Changes in the p210^Bcr-Abl^ protein levels treated with ASFE or imatinib (IM). K-562 cells were treated with (a) ASFE (2 *μ*g/mL) or (b) IM (2 *μ*mol) for increasing time periods (0–16 h). The p210^Bcr-Abl^ protein steady-state levels in the total lysates were analyzed by western blotting. Relative protein levels versus controls (beta-actin) were determined by densitometry. Data are the mean ± SD of three independent experiments (^*∗*^*P* < 0.05; ^*∗∗*^*P* < 0.01).

ASFE or IM was used to treat K-562 cells, and the p210^Bcr-Abl^ protein steady-state levels in the total lysate were analyzed by western blotting (Figure 4). 2 *μ*mol IM was subjected based on previous studies [41]. The p210^Bcr-Abl^ protein levels were decreased after treatment with ASFE or IM on K-562 cells in a time-dependent manner (^*∗*^*P* < 0.05; ^*∗∗*^*P* < 0.01).

### 3.7. ASFE Enhanced IM-Induced Destabilization of p210^*Bcr-Abl*^

**Figure 5:**
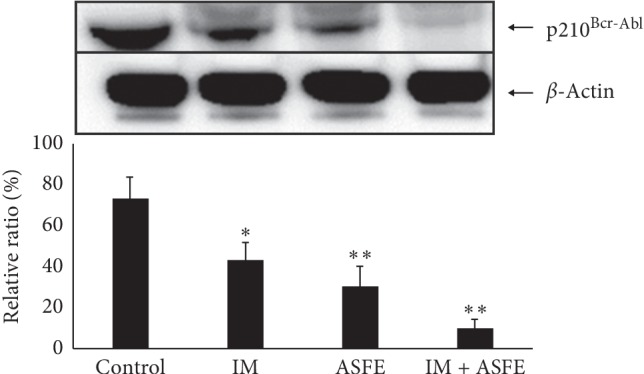
ASFE + IM combined therapy led to a decrease of p210^Bcr-Abl^. K-562 cells were treated with IM (2 *μ*mol), ASFE (2 *μ*g/mL), and IM + ASFE combination for 12 h at 37°C. The p210^Bcr-Abl^ protein levels in the total lysate were analyzed by western blotting (^*∗*^*P* < 0.05; ^*∗∗*^*P* < 0.01).

Figure 5 shows that when both ASFE and IM were added together to K-562 cells, the level of p210^Bcr-Abl^ was diminished compared to that of the lanes containing K-562 cells after treatment with IM or ASFE, indicating that the combined treatment of K-562 cells by ASFE and IM was more potent than each one separately.

### 3.8. Assessment of ASFE- and IM-Inducing Cell Death Mechanisms on K-562 Cells

**Figure 6:**
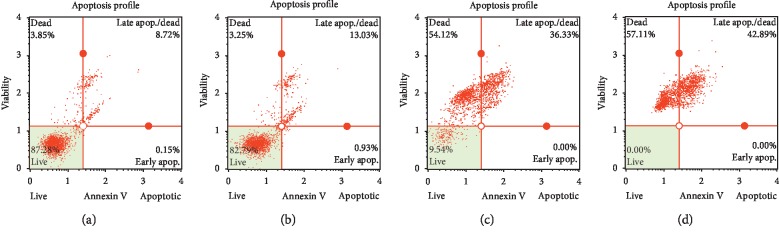
Apoptosis assessment of the effects of IM, ASFE, and IM + ASFE on K-562 cells. After 16 h of treatment, the cells were stained with annexin V/7-AAD reagent and cytometric analysis was performed using a MUSE™ cell analyzer. (a) Control, (b) IM, (c) ASFE, (d) IM + ASFE.

Apoptosis, necrosis, and autophagy are well-known cell death mechanisms. To observe apoptosis and necrosis, we conducted flow cytometry using annexin V and 7-AAD staining. After 16 h treatment of IM, ASFE, or IM + ASFE, the cells were stained with both annexin V and 7-AAD. The results showed that, in the control, >85% of the cells survived; the cell population decreased to 82.79% for the IM, 9.54% for the ASFE, and 0% for the IM + ASFE. The late apoptotic/necrotic cell population increased to 13.03% (IM), 36.33% (ASFE), and 42.89% (ASFE + IM). The early apoptotic cell population increased to 0.93% (IM), 0.00% (ASFE), and 0.00% (ASFE + IM) (Figure 6). The cell populations of late apoptosis/necrosis significantly increased, but increased cell population during early apoptosis was rare. Based on these results, we postulated that K-562 cells were involved in an alternate pathway and were not involved in apoptosis. These results also showed that K-562 cell killing was significant after treatment with ASFE.

## 4. Discussion

Traditionally, ASF has been used in Chinese medicine for the treatment of skin inflammation, stomach aches, and rheumatic pain [1]. For decades, herbal medicine has been used as an adjunct in chemotherapy for cancer treatment, demonstrating their synergistic roles in enhancing efficacy, ameliorating side effects, and reducing drug resistance [42]. However, there have been few scientific studies describing the anticancer effects of ASF.

CML is a clonal myeloproliferative disorder characterized by the presence of a constitutive tyrosine kinase activity of the fusion oncogene, *Bcr-Abl* [11]. Bcr-Abl protein induces cellular transformation by activating the signaling molecules, STAT5 and Akt [43]. As a first-line treatment, TKI-targeted Bcr-Abl was expected to treat CML effectively, but it has shown drug resistance, so the next generation of drugs from imatinib has been required [44]. Bcr-Abl T315I is the most frequent resistant mutation to tyrosine kinase inhibitors [45]. Following imatinib, the second-generation inhibitors such as dasatinib, nilotinib, and bosutinib and third-generation inhibitor such as ponatinib have been used to treat resistant cases of CML. Ponatinib has been developed to overcome the T315I mutation. The next candidates of tyrosine kinase inhibitors, including bafetinib, danusertib, rebastinib, tozasertib, HG-7-85-01, GNF-2, and 1,3,4-thiadiazole derivatives are presently undergoing approval as the next generation of therapeutic agents [46]. However, the issue of drug resistance is a rapidly increasing problem, and alternatives that are less toxic are urgently needed. Accordingly, this study aimed at providing the potential for exploring new therapies that are less harmful and more effective.

In the present study, we used a systemic pharmacological approach, filtered active compounds of ASF, constructed the compound-target network, and screened cancer-related compounds of ASF. ASF was found to contain 49 identified compounds, and 33 compounds were active in biological pathways related to cancers. Moreover, 10 of the 33 were classified as being associated with CML. Additionally, the compound-target network showed multiple compounds and multiple targeting characteristics of ASF. Multiple compounds and multiple targeting actions of other herbal medicines such as licorice [47], Xiao-Chaihu Decoction, Da-Chaihu Decoction [48], Pulsatillae Radix, Baekduong-tang [49, 50], and Bulsusan [51] have also been studied. Based on our results, we propose that ASF is a potential antileukemic agent.

Experiments were conducted to confirm this possibility. First, the active components of ASF were extracted. According to previous studies, the extraction of ASF could be conducted using several extraction techniques such as hydrodistillation, steam distillation, solvent extraction, supercritical fluid CO_2_ extraction, hydrodistillation-headspace solvent microextraction, and microwave-assisted extraction [52, 53]. In our study, a combination of solvent extraction and sonication, followed by pressured gas blowing concentration, was used. To obtain the maximum extraction ASF, methanol was used as a solvent [54], followed by sonication to increase the extraction efficiency. The 4-prophenylanisole was one of the main compounds of ASF and accounted for 94% of the essential oil [55]. Another major component of ASF was 4-anisaldehyde, which is used as a food fragrance and reported to have antifungal and saliva-enhancing effects [56]. In this study, the main compounds of 4-prophenylanisole and 4-anisaldehyde were identified in the ASFE using HPLC.

The ASFE and ASFE + IM combination showed potent antileukemic effects by decreasing the p210^Bcr-Abl^ levels. Because leukemic stem cells do not respond to TKI, when the drug supply is stopped, the cells can continuously multiply to allow the disease to persist. Moreover, the combination of treatment targets results, more efficiently, in alternative surviving mechanisms of cancer cells such as autophagy [16].

**Figure 7:**
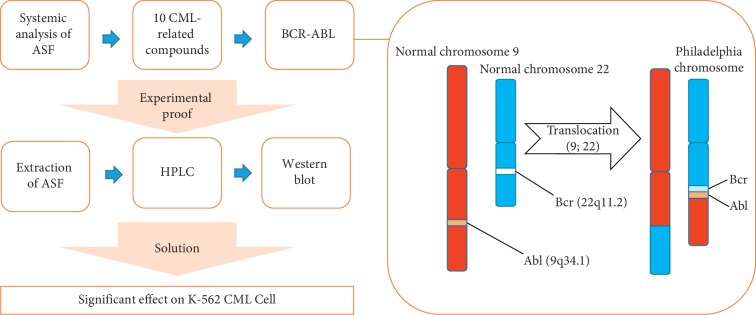
A workflow scheme illustrating the hypothesis that compounds of ASF having antileukemic potential can be identified using systematic pharmacological approaches; the experimental proof was that ASF was effective in killing of K-562 chronic myeloid leukemia cells.

Our results showed that ASFE combined with IM resulted in the significant death of K-562 cells. The results suggested the feasibility of further research to identify cell death mechanisms following a decrease in Bcr-Abl. In addition, our next phase of research will be to verify the effects of 10 CML-related compounds as well as two representative compounds of ASF, 4-anisaldehyde and 4-prophenylanisole, on CML. The workflow scheme of our study is summarized in Figure 7.

## 5. Conclusion

We have identified 10 CML-related compounds of ASF using systematic pharmacological analyses. The ASF extract significantly induced cell death of K-562 cells. In addition, combined treatment with ASFE and IM led to a significant decrease of Bcr-Abl protein levels. The results showed that ASF is a promising source of effective chemotherapeutic adjuvants and is a candidate, in combination with other chemotherapeutic drugs, for the treatment of CML patients, to ameliorate the side effects and overcome resistance. Although we do not know the exact mechanisms of the 10 compounds of ASF and their antileukemic effects, this study is the first to report the antileukemic effects of ASF on K-562 CML cells, which should prompt further mechanistic studies of these antileukemic compounds.

## Figures and Tables

**Table 1:** The 33 compounds and their related cancers.

Active compounds	Related cancers
C09628	Bladder cancer Breast cancer Cancer, unspecific Colorectal cancer Genitourinary tumors Lung cancer Oropharyngeal squamous cell carcinoma Prostate cancer Renal cell carcinoma

Kaempferol	Prostate cancer Adrenocorticotropic hormone-secreting pituitary tumors Bladder cancer Pancreatic cancer Renal cell carcinoma Testicular cancer Thyroid follicular carcinoma Breast cancer Colorectal cancer Genitourinary tumors Lung cancer Head and neck cancer Malignant mesothelioma Meningioma Oropharyngeal squamous cell carcinoma Chronic myelogenous leukemia (CML) Gastrointestinal stromal tumors (GIST) Hematological malignancies HER2-positive Metastatic breast cancer Melanoma Multiple myeloma Non-small-cell lung cancer Ovarian cancer Refractory hematological malignancies Solid tumors Malignancies Gliomas Colon cancer Acute promyelocytic leukemia Chronic lymphocytic leukemia Prostate cancer (hormone-refractory) Waldenstrom's macroglobulinemia Solid tumor Cancer (multidrug-resistant) Chondrosarcoma Hormone-refractory prostate cancer Kaposi's sarcoma Breast neoplasms Colorectal neoplasms Ovarian neoplasms Urinary bladder neoplasms Bronchiolar carcinoma Cervical cancer Rectal neoplasms Chronic myeloid leukemia Gastrointestinal cancers Urological cancers Non-small-cell lung carcinoma Gastrointestinal cancers Gastrointestinal neoplasms Head and neck neoplasms Neoplasms Precursor cell Lymphoblastic leukemia-lymphoma


ZINC02040970	Bladder cancer Breast cancer Cancer, unspecific Carcinoma in situ unspecified Colorectal cancer Genitourinary tumors Lung cancer Oropharyngeal squamous cell carcinoma Prostate cancer Renal cell carcinoma

(1R,5R,7S)-4,7-Dimethyl-7-(4-methylpent-3-enyl)bicyclo[3.1.1]hept-3-ene	Bladder cancer Breast cancer Cancer, unspecific Carcinoma in situ, unspecified Colorectal cancer Genitourinary tumors Lung cancer Malignant mesothelioma Oropharyngeal squamous cell carcinoma Prostate cancer Renal cell carcinoma

1,8-Cineole	Colon cancer Bladder cancer Breast cancer Cancer, unspecific Carcinoma in situ, unspecified Colorectal cancer Genitourinary tumors Lung cancer Oropharyngeal squamous cell carcinoma Prostate cancer

3-O-Feruloylquinic acid	Bladder cancer Breast cancer Cancer, unspecific Carcinoma in situ, unspecified Colorectal cancer Genitourinary tumors Lung cancer Malignant mesothelioma Oropharyngeal squamous cell carcinoma Prostate cancer Renal cell carcinoma Pancreatic cancer Tumors

Anisketone	Gliomas Bladder cancer Breast cancer Cancer, unspecific Carcinoma in situ, unspecified Colorectal cancer Genitourinary tumors Lung cancer Malignant mesothelioma Meningioma Oropharyngeal squamous cell carcinoma Prostate cancer Renal cell carcinoma Colon cancer Pancreatic cancer

Copaene	Bladder cancer Breast cancer Cancer, unspecific Carcinoma in situ, unspecified Colorectal cancer Genitourinary tumors Lung cancer Malignant mesothelioma Meningioma Oropharyngeal squamous cell carcinoma Prostate cancer Renal cell carcinoma

Terpilene	Bladder cancer Breast cancer Cancer, unspecific Carcinoma in situ, unspecified Colorectal cancer Genitourinary tumors Lung cancer Malignant mesothelioma Meningioma Oesophageal cancer Oropharyngeal squamous cell carcinoma Pathological angiogenesis Gliomas Renal cell carcinoma Leukemia, Myeloid malignancies Prostate cancer Solid tumors

Guaiene	Bladder cancer Breast cancer Cancer, unspecific Carcinoma in situ, unspecified Colorectal cancer Genitourinary tumors Lung cancer Malignant mesothelioma Meningioma Oropharyngeal squamous cell carcinoma Prostate cancer Renal cell carcinoma

Salicylic acid	Bladder cancer Breast cancer Cancer, unspecific Carcinoma in situ, unspecified Carpal tunnel syndrome Colorectal cancer Genitourinary tumors Lung cancer Malignant mesothelioma Meningioma Oropharyngeal squamous cell carcinoma Prostate cancer Renal cell carcinoma Bronchiolar carcinoma Cervical cancer Chronic myeloid leukemia Gastrointestinal cancers Pancreatic cancer Urological cancers Leukemia, myeloid, Acute Endometrial carcinoma Leukemia, unspecified Mesothelioma Tumors Precursor Cell lymphoblastic Leukemia-lymphoma Breast neoplasms Non-small-cell lung carcinoma Colorectal neoplasms Gastrointestinal neoplasms Head and neck neoplasms Neoplasms Ovarian neoplasms Rectal neoplasms

(Z)-Caryophyllene	Bladder cancer Breast cancer Cancer, unspecific Carcinoma in situ, unspecified Colorectal cancer Genitourinary tumors Lung cancer Malignant mesothelioma Meningioma Oropharyngeal squamous cell carcinoma Prostate cancer Renal cell carcinoma

(*R*)-Linalool	Bladder cancer Breast cancer Cancer, unspecific Carcinoma in situ, unspecified Colorectal cancer Genitourinary tumors Lung cancer Malignant mesothelioma Meningioma Oropharyngeal squamous cell carcinoma Prostate cancer Renal cell carcinoma Acute promyelocytic leukemia Chronic myelogenous leukemia (CML) Gastrointestinal stromal tumors (GIST) Hematological malignancies HER2-positive Metastatic breast cancer Melanoma Multiple myeloma Non-small-cell lung cancer Ovarian cancer Refractory hematological malignancies Solid tumors

Mairin	Breast cancer

Honokiol	Gliomas Breast cancer Prostate cancer Endocrine independent cancer Adrenocorticotropic hormone-secreting pituitary tumors Pancreatic cancer Bladder cancer Renal cell carcinoma Testicular cancer Thyroid follicular carcinoma Solid tumors Cancer, unspecific Carcinoma in situ, unspecified Colorectal cancer Genitourinary tumors Lung cancer Malignant mesothelioma Meningioma Refractory hematological malignancies Oropharyngeal squamous cell carcinoma Chronic myelogenous leukemia (CML) Malignancies Multiple myeloma Gastrointestinal stromal tumors (GIST) Hematological malignancies HER2-positive Metastatic breast cancer Melanoma Non-small-cell lung cancer Ovarian cancer Acute lymphoblastic leukemia (ALL) Acute myeloid leukemia (AML) Advanced solid tumors B-cell malignancies Chronic lymphocytic leukemia (CLL) Hepatocellular carcinoma (HCC) Nasopharyngeal cancer (NPC) Non-Hodgkin's lymphoma leukemia Myeloid Oesophageal cancer

Hemo-sol	Bladder cancer Breast cancer Cancer, unspecific Carcinoma in situ, unspecified Colorectal cancer Genitourinary tumors Lung cancer Malignant mesothelioma Meningioma Oropharyngeal squamous cell carcinoma Prostate cancer

(1S,5S)-1-Isopropyl-4-methylenebicyclo[3.1.0]hexane	Bladder cancer Breast cancer Cancer, unspecific Carcinoma in situ, unspecified Colorectal cancer Genitourinary tumors Lung cancer Malignant mesothelioma Meningioma Oropharyngeal squamous cell carcinoma Prostate cancer Renal cell carcinoma

CHEBI:7	Chronic myeloid leukemia Breast cancer Bladder cancer Cancer, unspecific Carcinoma in situ, unspecified Colorectal cancer Genitourinary tumors Lung cancer Malignant mesothelioma Meningioma Oropharyngeal squamous cell carcinoma Prostate cancer Renal cell carcinoma Acute promyelocytic leukemia

Luteolin	Prostate cancer Bladder cancer Breast cancer Cancer, unspecific Carcinoma in situ, unspecified Colorectal cancer Genitourinary tumors Lung cancer Malignant mesothelioma Meningioma Oropharyngeal squamous cell carcinoma Renal cell carcinoma Chronic myelogenous leukemia (CML) Gastrointestinal stromal tumors (GIST) Hematological malignancies HER2-positive Metastatic breast cancer Melanoma Multiple myeloma Non-small-cell lung cancer Ovarian cancer Refractory hematological malignancies Solid tumors Malignancies Head and neck tumors Pancreatic cancer Solid tumor Squamous cell carcinoma tumors Colorectal neoplasms Brain cancer Hepatocellular carcinoma Hormone-refractory prostate cancer Kaposi's sarcoma Cancer (multidrug-resistant) Kidney cancer Chondrosarcoma Breast neoplasms Non-small-cell lung carcinoma Gastrointestinal neoplasms Head and neck neoplasms Neoplasms Ovarian neoplasms Precursor cell Lymphoblastic leukemia-lymphoma Rectal neoplasms Gastric cancer Prostate cancer (metastatic) Small-cell lung cancer

HCI	Gliomas Bladder cancer Breast cancer Cancer, unspecific Carcinoma in situ, unspecified Colorectal cancer Genitourinary tumors Lung cancer Malignant mesothelioma Meningioma Oropharyngeal squamous cell carcinoma Prostate cancer Renal cell carcinoma Malignancies

Beta-selinene	Bladder cancer Breast cancer Cancer, unspecific Carcinoma in situ, unspecified Colorectal cancer Genitourinary tumors Lung cancer Malignant mesothelioma Meningioma Oropharyngeal squamous cell carcinoma Prostate cancer Renal cell carcinoma

2-Methyl-*N*-phenylmaleimide	Cancer, unspecific Tumors Bladder cancer Breast cancer Carcinoma in situ, unspecified Colorectal cancer Genitourinary tumors Lung cancer Prostate cancer Renal cell carcinoma Pancreatic cancer Chronic myeloid leukemia Oesophageal cancer

2-(3,4-Dihydroxyphenyl)-5,7-dihydroxy-3-[(2R,3R,4S,5R)-3,4,5-trihydroxytetrahydropyran-2-yl]oxy-chromone	Bladder cancer Breast cancer Cancer, unspecific Carcinoma in situ, unspecified Colorectal cancer Genitourinary tumors Lung cancer Malignant mesothelioma Meningioma Prostate cancer Meningioma Renal cell carcinoma Oropharyngeal squamous cell carcinoma Acute promyelocytic leukemia

Anethole	Glaucoma Breast cancer Cancer (multidrug-resistant) Melanoma

(+)-Catechin	Breast cancer Endocrine independent cancer Bladder cancer Cancer, unspecific Colorectal cancer Genitourinary tumors Lung cancer Malignant mesothelioma Meningioma Oropharyngeal squamous cell carcinoma Prostate cancer Renal cell carcinoma Chronic myelogenous leukemia (CML) Refractory hematological malignancies Gastrointestinal stromal tumors (GIST) Precursor cell Lymphoblastic leukemia-lymphoma Hematological malignancies HER2-positive Metastatic breast cancer Melanoma Non-small-cell lung cancer Ovarian cancer Solid tumors

Shikimic acid	Bladder cancer Breast cancer Cancer, unspecific Carcinoma in situ, unspecified Colorectal cancer Genitourinary tumors Lung cancer Malignant mesothelioma Meningioma Oropharyngeal squamous cell carcinoma Prostate cancer Renal cell carcinoma

Astragalin	Bladder cancer Breast cancer Cancer, unspecific Carcinoma in situ, unspecified Colorectal cancer Lung cancer Genitourinary tumors Prostate cancer Malignant mesothelioma Meningioma Oropharyngeal squamous cell carcinoma Renal cell carcinoma Acute promyelocytic leukemia Chronic myelogenous leukemia (CML) Gastrointestinal stromal tumors (GIST) Hematological malignancies HER2-positive Metastatic breast cancer Melanoma Multiple myeloma Non-small-cell lung cancer Ovarian cancer Refractory hematological malignancies Solid tumors Gliomas Malignancies

Cis-beta-farnesene	Bladder cancer Breast cancer Cancer, unspecific Carcinoma in situ, unspecified Colorectal cancer Meningioma Genitourinary tumors Malignant mesothelioma Lung cancer Oropharyngeal squamous cell carcinoma Prostate cancer Renal cell carcinoma

5,7-Dihydroxy-2-(4-hydroxyphenyl)-3-[(2R,3R,4S,5R,6R)-3,4,5-trihydroxy-6-(hydroxymethyl)oxan-2-yl]oxychromen-4-one	Acute promyelocytic leukemia Cancer, unspecific

Beta-bisabolene	Bladder cancer Prostate cancer Breast cancer Cancer, unspecific Renal cell carcinoma Colorectal cancer Carcinoma in situ, unspecified Genitourinary tumors Lung cancer Malignant mesothelioma Meningioma Oropharyngeal squamous cell carcinoma

Quercetin	Prostate cancer Bladder cancer Pancreatic cancer Renal cell carcinoma Adrenocorticotropic hormone-secreting pituitary tumors Testicular cancer Thyroid follicular carcinoma Breast cancer Cancer, unspecific Carcinoma in situ, unspecified Colorectal cancer Malignant mesothelioma Meningioma Urinary bladder neoplasms Genitourinary tumors Lung cancer Melanoma Multiple myeloma Non-small-cell lung cancer Oropharyngeal squamous cell carcinoma Ovarian cancer Chronic myelogenous leukemia (CML) Solid tumors Hematological malignancies Malignancies Gastrointestinal stromal tumors (GIST) Gliomas HER2-positive Metastatic breast cancer Brain cancer Colon cancer Solid tumor Refractory hematological malignancies Tumor Acute promyelocytic leukemia Kaposi's sarcoma Head and neck tumors Rectal neoplasms Cancer (multidrug-resistant) Kidney cancer Squamous cell carcinoma Colorectal neoplasms Breast neoplasms Ovarian neoplasms Chronic lymphocytic leukemia Hormone-refractory prostate cancer Waldenstrom's macroglobulinemia Endometrial neoplasms Hepatocellular carcinoma Chondrosarcoma Bronchiolar carcinoma Urological cancers Non-small-cell lung carcinoma Gastrointestinal neoplasms Head and neck neoplasms Cervical cancer Chronic myeloid leukemia Gastrointestinal cancers Neoplasms Precursor cell lymphoblastic leukemia-lymphoma

Terragon	Cancer, unspecific Bladder cancer Breast cancer Carcinoma in situ, unspecified Colorectal cancer Genitourinary tumors Lung cancer Malignant mesothelioma Meningioma Oropharyngeal squamous cell carcinoma Prostate cancer Renal cell carcinoma

Humulene	Bladder cancer Breast cancer Cancer, unspecific Carcinoma in situ, unspecified Colorectal cancer Genitourinary tumors Lung cancer Malignant mesothelioma Meningioma Prostate cancer Solid tumor

**Table 2:** The 10 CML-related compounds of Anisi stellati fructus.

Molecule name	MW	OB (%)	Caco-2	DL	HL
Astragalin	448.41	14.03	−1.34	0.74	N/A
CHEBI:7	136.26	45.2	1.84	0.04	11.44
(+)-Catechin	290.29	54.83	−0.03	0.24	0.61
2-Methyl-*N*-phenylmaleimide	187.21	87.36	0.77	0.06	2.93
Kaempferol	286.25	41.88	0.26	0.24	14.74
Honokiol	266.36	60.67	1.43	0.15	2.88
Luteolin	286.25	36.16	0.19	0.25	15.94
Salicylic acid	138.13	32.13	0.63	0.03	12
(*R*)-Linalool	154.28	39.8	1.33	0.02	6.48
Quercetin	302.25	46.43	0.05	0.28	14.4

MW: molecular weight; OB: oral bioavailability; Caco-2: Caco-2 permeability; DL-2: drug likeness; HL: drug half-life.

## Data Availability

All data used to support the findings of this study are included within the article.
